# Pale-Green Phenotype of *atl31* *atl6* Double Mutant Leaves Is Caused by Disruption of 5-Aminolevulinic Acid Biosynthesis in *Arabidopsis thaliana*

**DOI:** 10.1371/journal.pone.0117662

**Published:** 2015-02-23

**Authors:** Shugo Maekawa, Atsushi Takabayashi, Thais Huarancca Reyes, Hiroko Yamamoto, Ayumi Tanaka, Takeo Sato, Junji Yamaguchi

**Affiliations:** 1 Faculty of Science and Graduate School of Life Science, Hokkaido University, Sapporo, Hokkaido, Japan; 2 Institute of Low Temperature Science, Hokkaido University, Sapporo, Hokkaido, Japan; Universidade Federal de Vicosa, BRAZIL

## Abstract

Arabidopsis ubiquitin ligases ATL31 and homologue ATL6 control the carbon/nitrogen nutrient and pathogen responses. A mutant with the loss-of-function of both *atl31* and *atl6* developed light intensity-dependent pale-green true leaves, whereas the single knockout mutants did not. Plastid ultrastructure and Blue Native-PAGE analyses revealed that pale-green leaves contain abnormal plastid structure with highly reduced levels of thylakoid proteins. In contrast, the pale-green leaves of the *atl31/atl6* mutant showed normal Fv/Fm. In the pale-green leaves of the *atl31/atl6*, the expression of *HEMA1*, which encodes the key enzyme for 5-aminolevulinic acid synthesis, the rate-limiting step in chlorophyll biosynthesis, was markedly down-regulated. The expression of key transcription factor *GLK1*, which directly promotes *HEMA1* transcription, was also significantly decreased in *atl31/atl6* mutant. Finally, application of 5-aminolevulinic acid to the *atl31/atl6* mutants resulted in recovery to a green phenotype. Taken together, these findings indicate that the 5-aminolevulinic acid biosynthesis step was inhibited through the down-regulation of chlorophyll biosynthesis-related genes in the pale-green leaves of *atl31/atl6* mutant.

## Introduction

Chlorophyll (Chl) biosynthesis is regulated primarily at the level of 5-aminolevulinic acid (ALA) synthesis. In higher plants, ALA is synthesized from glutamate in the plastid in three enzymatic steps. In the first step, the enzyme glutamyl-tRNA synthetase aminoacylates tRNA^Glu^ with glutamate, generating the substrate for plastid translation and tetrapyrrole biosynthesis. In the following steps, tRNA^Glu^ is reduced by glutamyl-tRNA reductase (GluTR) to glutamate-1-semialdehyde, which is converted to ALA by glutamate-1-semialdehyde aminotransferase [1,2].

Of the three enzymes responsible for ALA synthesis, GluTR is regarded as the main target of regulatory mechanisms [3]. The transcription and activity of GluTR is controlled by light, an endogenous clock, cytokinin and development [2–5]. In Arabidopsis, three *HEMA* genes encode GluTR. *HEMA3* is considered a pseudogene [6], whereas *HEMA1* and *HEMA2* encode proteins that are 81% identical at the amino acid level [7,8]. Since *HEMA1* is highly expressed in photosynthetic tissues of leaves and stems, whereas *HEMA2* is constitutively expressed at low levels in all tissues, *HEMA1* is considered the dominant form of GluTR for Chl biosynthesis [9,10]. RNA interference (RNAi)-induced down-regulation of *HEMA1* expression in Arabidopsis and tobacco resulted in a reduction in plant Chl [5,11]. Furthermore, a *hema1* mutant showed a yellowish phenotype, due to a drastically reduced level of Chl with severe growth retardation [12]. Thus, regulating *HEMA1* is essential for Chl biosynthesis.

Genes in the Arabidopsis Tóxicos en Levadura (ATL) family encode plant-specific putative RING-type ubiquitin ligases with transmembrane domains. In Arabidopsis, the ATL family is composed of 91 members [13,14]. ATL31 and its closest homolog ATL6 are membrane-associated ubiquitin ligases, shown to be involved in the carbon/nitrogen (C/N) response by regulating the stability of 14–3–3 proteins through their ubiquitination activity [15–17]. Plants overexpressing full-length *ATL31* or *ATL6* (*35S-ATL31* and *35S-ATL6)* were insensitive to high C/N stress conditions, whereas the single knockout mutants *atl31–1* and *atl6–1*, as well as an *atl31–1/atl6–1* double knockout mutant showed increased sensitivity to high C/N stress conditions [15,18]. ATL31 and ATL6 are also involved in the plant immune response. *35S-ATL31* showed enhanced resistance to the bacterial pathogen *Pseudomonas syringae* pv. *tomato* DC3000 and powdery mildew *Bgh*. [18,19]. Moreover, the double mutant, but not the single mutants, showed increased susceptibility to *Pst*. DC3000. In contrast, the double mutant and wild-type (WT) showed comparable resistance to *Bgh* entry [18,19]. In addition, the true leaves of the *atl31–1/atl6–1* double mutant had a pale-green phenotype during its early developmental stage [15]. However, the mechanism underlying this pale-green phenotype remains unknown.

In this present study, we found that this phenotype was dependent on light intensity. Under low light conditions, the double mutant appeared similar to WT, whereas under medium light conditions, the double mutant showed a decreased level of Chl, an abnormal plastid structure and reduced levels of plastid proteins on their true leaves. Expression analyses revealed that the expression of *HEMA1* and *GLK1*, which encodes key transcription factor for Chl biosynthesis related genes including *HEMA1*, were markedly lower in the pale-green true leaves of the double mutant than that in WT plants. Application of exogenous ALA could restore the pale-green phenotype of the double mutant to a green phenotype, bypassing the reduction of HEMA1 function. These findings indicate the involvement of ATL31 and ATL6 to Chl biosynthesis by controlling 5-aminolevulinic acid biosynthesis step through the down-regulation of *HEMA1* gene.

## Materials and Methods

### Plant materials and growth conditions

Columbia-0 was used as WT Arabidopsis. For germination, seeds were surface sterilized and placed on Murashige and Skoog medium supplemented with 20 g l^-1^ sucrose formed by 4 g l^-1^ gellan gum. After 48 h cold treatment to synchronize germination, seeds were grown at 22°C and 50% relative humidity under a 16/8 h light/dark cycle under indicated conditions of light intensity. The *35S-ATL31*, *atl31–1*, *atl6–1*, *atl31–1/atl6–1* [15], and *35S-ATL6* [18] mutants were obtained as described, and *atl6–2* (SALK_134489) [20] was obtained from the Arabidopsis Biological Resource Center.

### Measurement of chlorophyll and Fv/Fm

Chl content and the Chl a/b ratio were determined as described [21]. The maximal photochemical efficiency of PSII (Fv/Fm) was measured with a PAM-2000 chlorophyll fluorometer (Walz, Germany) as described [22].

### Molecular cloning

To generate an RNAi transgenic plant targeting *ATL31* and *ATL6*, a fragment of complementary DNA encoding a truncated ATL6 was amplified by PCR using pENTRATL6 [18] as the template and the primers shown in S1 Table. The resulting product was cloned into the pENTR/D-TOPO vector (Life Technologies) to generate the plasmid pENTRATL6RNAi. The ATL6RNAi fragment was recombined into the pHellsgate12 transfer-DNA binary vector [23] according to the manufacturer’s directions (Life Technologies). All PCR products and inserts were verified by DNA sequencing.

### Gene expression analysis

RNA isolation, reverse transcription and PCR were performed as described [24]. RT-PCR was performed using normalized cDNA samples. PCR products were electrophoresed on agarose gels and visualized by ethidium bromide staining. Quantitative real-time PCR was performed with the SYBR Premix Ex Taq (TaKaRa) on an Applied Biosystems 7300 Real-Time PCR system (Applied Biosystems) according to the manufacturer’s instructions. All signals were normalized relative to the level of expression of *EF1α*, with relative gene expression calculated using the ΔΔCT method. The primers used are listed in S1 Table.

### Transmission electron microscopy

To assess plastid structure, first or second leaves from 2-week old WT and *atl31–1/atl6–1* plants were fixed in 1% (w/v) glutaraldehyde, 4% (w/v) paraformaldehyde in 0.05 M phosphate buffer, pH 7.4, for 2 h at room temperature, and washed several times with phosphate buffer. The samples were further fixed in 0.5% (w/v) osmium tetroxide, 0.15 M phosphate buffer, pH 7.4, for 2 h at room temperature, washed twice with phosphate buffer, and dehydrated using an acetone series prior to infiltration with Spurr’s resin (Polysciences, Inc.). Following polymerization at 70°C for 8 h, the ultra-thin sections were cut using an ultramicrotome (EM UC6, Leica Microsystems) and viewed under a transmission electron microscope (H-7650, Hitachi).

### Blue-Native PAGE, immunoblot analysis and CBB staining

BN-PAGE and immunoblot analyses were performed as described [25]. Total proteins were extracted from first or second true leaves of 2-week-old WT and *atl31–1/atl6–1*, with 8 mg of FW used for BN-PAGE and 0.5 mg FW for immunoblotting. For immunoblot analyses, the transferred proteins were detected with antibodies to PsbC (CP43) and PsaA/PsaB (CP1) [26] and with antibodies to PsbD and Lhcb1 (Agrisera, Sweden). For CBB staining, the proteins were visualized using EzStain AQua (ATTO).

## Results

### Double mutant *atl31/atl6* developed light intensity-dependent pale-green leaves

**Fig 1:**
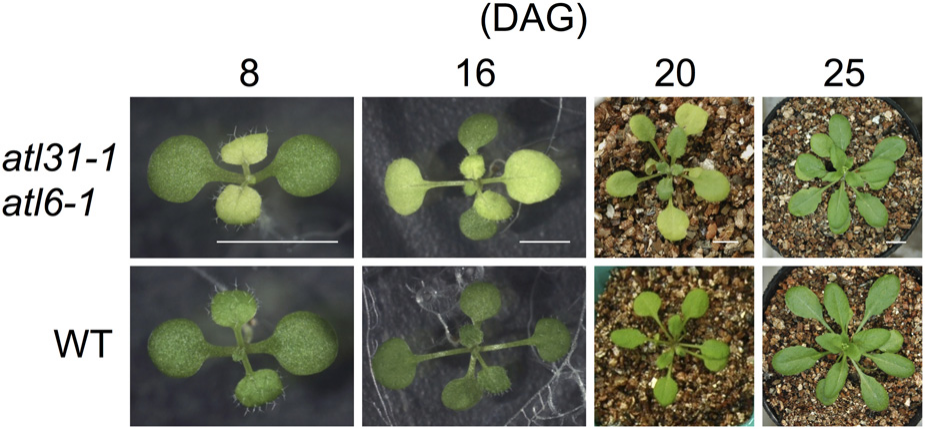
Manifestation of the pale-green phenotype in the *atl31–1/atl6–1* double mutant. Photographs of representative *atl31–1/atl6–1* and WT plants over time. Scale bar: 5 mm.

**Fig 2:**
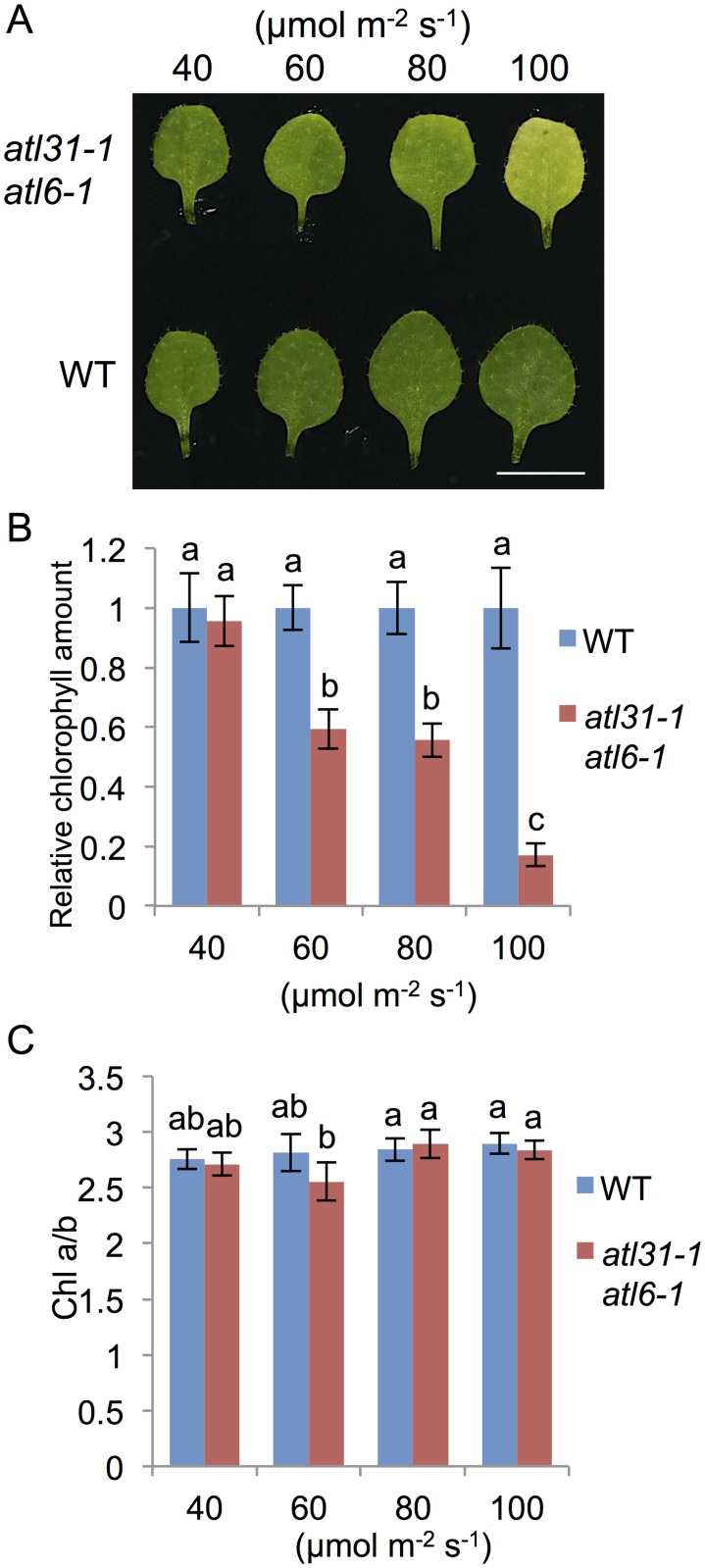
Dependence of the pale green phenotype of *atl31–1/atl6–1* double mutants on light intensity. WT and *atl31–1/atl6–1* plants were grown at the indicated light intensity for 2 weeks. (A) Photographs of representative first or second leaves of each plant. Scale bar: 5 mm. (B) and (C) Chl amount and Chl a/Chl b ratio (Chl a/b) in first or second leaves of each plant respectively. Error bars represent SD (n = 5). Statistical significance was determined by ANOVA, followed by post-hoc Tukey’s tests. Means that differed significantly (*P*<0.05) are indicated by different letters.

We previously found that the *atl31–1/atl6–1* double mutant had pale-green true leaves during early stages of development under normal growth conditions (MS medium, light/dark; 16 h/8 h, 100 μmol m^-2^ s^-1^), with cotyledons similar to those of WT (Fig. 1 and [15]). These pale-green leaves of mutant plants gradually turned green from the petiole to the leaf apex about 25 days after germination (Fig. 1), having green-colored leaves at the matured stage, similar to those of WT plants (S1A and S1B Fig.). Efforts to identify the key factor responsible for this phenotype in mutant plants found that the loss of pigment was dependent on light intensity (Fig. 2A and 2B). Under low light (LL) conditions (40 μmol m^-2^ s^-1^), the color and Chl contents of mutant true leaves were similar to those of WT. However, as light intensity increased, the chlorophyll contents of mutant leaves were decreased (Fig. 2A and 2B). The ratio of Chl a to Chl b (Chl a/b) in the mutant was similar to that of WT and independent of light intensity, with leaves showing a pale-green color under medium light (ML: 100 μmol m^-2^ s^-1^) conditions (Fig. 2C). In contrast to the double mutants, plants with single mutations in *atl31–1* or *atl6–1* and those overexpressing *ATL31* and *ATL6* did not develop any pale-green leaves even under ML conditions (S2 Fig.). To exclude the possibility that this pale-green phenotype is caused by the insertion of other T-DNA sequences, we generated another type of *atl31/atl6* double mutant, using different alleles of *atl6* mutants and RNAi. The double mutant of *atl31–1* and *atl6–2*, a null-mutant of *ATL6* (S3A and S3B Fig.), showed a pale-green phenotype similar to that of the *atl31–1/atl6–1* double mutant (S3C Fig.). Moreover, we generated double knock down transgenic plants for *atl31* and *atl6* by RNAi technique using the RNAi generating vector pHELLSGATE12 [23]. This construct produced a hairpin RNA containing approximately 300 bp of the ATL6 coding region, sharing 75% identity with ATL31. Plants targeted by *ATL31/ATL6* also had a pale-green phenotype (S3C and S3D Fig.). These findings indicated that a simultaneous loss of function of the *ATL31* and *ATL6* genes led to a light-dependent decrease in the amounts of Chl, with this decrease responsible for the pale-green phenotype.

### Plastids of *atl31/atl6* pale-green leaves had reduced inner membrane structure

**Fig 3:**
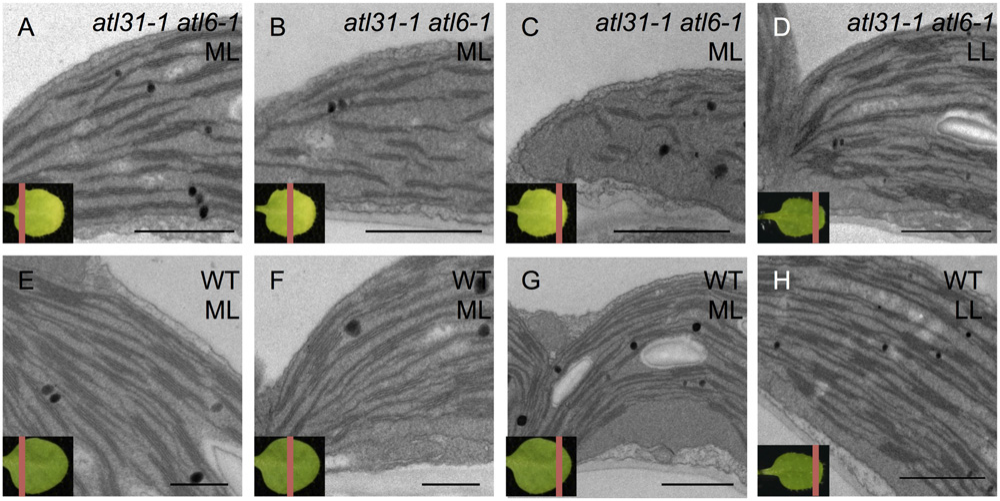
Plastid ultrastructure. Transmission electron microscopic images of first or second true leaves of 2-week old plants *atl31–1/atl6–1* (A-D) and WT (E-H) plants grown under ML (A-C and E-G) and LL (D and H) conditions. A red line on the left below representative leaves indicated the section of each sample. Scale bar: 1 μm.

To investigate the mechanism by which knockout of the *ATL31* and *ATL6* genes resulted in a pale-green phenotype, we examined plastid ultrastructure by transmission electron microscopy (TEM). Since the pale-green phenotype was gradually recovered from the petiole to the leaf apex (Fig. 1), we separated a pale-green leaf into 3 sections along the proximal/distal axis to observe the recovering of color of the pale-green leaves (Fig. 3). Under ML conditions, WT plants generated normal crescent-shaped chloroplasts containing plastoglobules, starch grains and well-developed thylakoid structures including stromal thylakoids and grana stacks across sections of the entire leaf (Fig. 3E–3G). In the same way, the bottom section of an *atl31–1/atl6–1* leaf plastid also showed a structure similar to that of WT under ML conditions (Fig. 3A). However, middle section of *atl31–1/atl6–1* leaf plastids was found to contain reduced inner membrane structure (Fig. 3B). Furthermore, leaf plastids in the apex section of *atl31/atl6* showed almost total loss of stromal thylakoids and reduced grana stacks (Fig. 3C). These features of the mutant plastid did not resemble the properties of immature leaves or the process of greening or the other pale-green mutants [27–31]. Under LL conditions, however, the structures of the *atl31–1/atl6–1* plastids were similar to that of WT even in the leaf apex section (Fig. 3D and 3H) which is consistent with the same Chl content in WT and *atl31–1/atl6–1* (Fig. 2). Lower-magnification views were shown in S4 Fig.. These results suggest that plastid biogenesis in the *atl31–1/atl6–1* double mutants was impaired only during early leaf development and in a light intensity-dependent manner.

### Double mutant *atl31/atl6* pale-green leaves had reduced levels of thylakoid proteins

**Fig 4:**
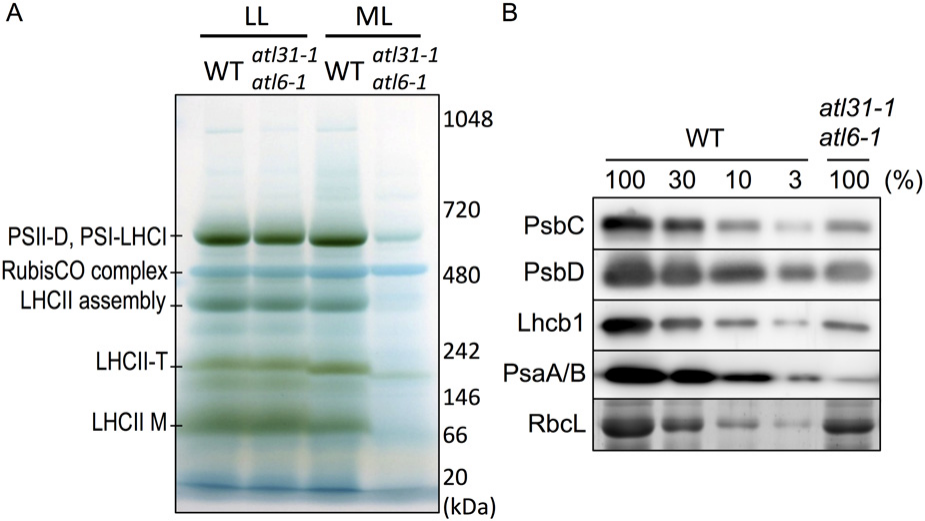
BN-PAGE and immunoblot analyses. (A) Separation and identification of the pigment-protein complexes in first and second true leaves of 2-week old WT and *atl31–1/atl6–1* plants grown under ML and LL conditions. The BN-PAGE gels were destained with 20% MeOH and 7% acetic acid to clarify the bands of the pigment-protein complexes. The PSII dimer (PSII-D), PSI-LHCI supercomplex, RubisCO complex, CP29-CP24-LHCII trimer (LHCII assembly), LHCII trimer (LHCII-T) and LHCII monomer (LHCII-M) were visualized in the BN-gel. Proteins were identified as described [25,32,33]. The positions of the molecular markers are indicated on the right. (B) Immunoblot analyses and CBB staining of the plastid proteins in first and second true leaves of 2-week-old WT and *atl31–1/atl6–1* plants grown under ML conditions. Total leaf extracts were loaded onto 14% SDS—PAGE gels containing 4M urea. Immunoblot analyses were performed using antibodies to PsbC, PsbD, Lhcb1 and PsaA/B, whereas RbcL was visualized by CBB staining. A dilution series of the WT samples is indicated in percentage.

Since the structures of the plastids in *atl31–1/atl6–1* resulted in abnormal leaves under ML conditions, we investigated Chl-protein complexes by blue native-polyacrylamide gel electrophoresis (BN-PAGE). Total proteins from the leaves of 2-week-old WT and *atl31–1/atl6–1* were separated by BN-PAGE with 4–13% linear gradient gels followed by CBB staining. Although the leaves of the double mutant (*atl31–1/atl6–1*) had normal levels of expression of Chl-protein complexes, comparable with those of WT under LL conditions, the mutant leaves had markedly reduced levels of Chl-protein complexes under ML conditions, despite these plants having comparable amounts of RubisCO (Fig. 4A).

To confirm the decrease in Chl-binding proteins in *atl31–1/atl6–1* under ML conditions, we performed SDS-PAGE followed by Immunoblotting and CBB staining analyses on these samples (Fig. 4A). We found that the PSII complex proteins PsbC and PsbD, and Lhcb1, a major LHCII complex protein, were markedly reduced in approximately 10% of that in WT (Fig. 4B). PsaA/B, which are PSI complex protein, were drastically reduced, to approximately 1% of that in WT (Fig. 4B). In contrast, the RbcL level of *atl31–1/atl6–1* was normal or somewhat moderately decreased compared with WT (Fig. 4B). Since PSII and LHCII are dominant in the grana stack and PSI is dominant in stroma thylakoids [34,35], the results of immunoblot analyses were consistent with the plastid ultrastructure of pale-green leaves, which showed loss of stromal thylakoids with reduced grana stacks (Fig. 3).

**Fig 5:**
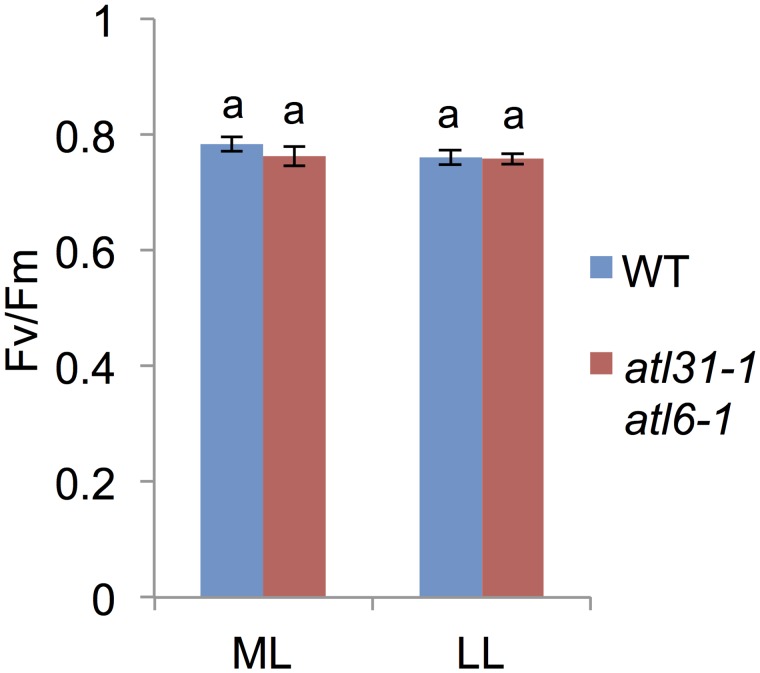
Fv/Fm measurement. Effect of light conditions on the Fv/Fm of first or second true leaves of 2-week-old WT and *atl31–1/atl6–1* plants grown under ML or LL conditions. Error bars represent SD (n = 5). Statistical significance was determined by ANOVA, followed by post-hoc Tukey’s tests. Means that differed significantly (*P*<0.05) are indicated by different letters.

To determine whether the ratio of photo-damaged PSII was increased in the *atl31–1/atl6–1* double mutant, we measured Fv/Fm, but found no significant difference between these plants and WT, even when grown under ML conditions (Fig. 5). This result, showing that the ratio of functional to total PSII in *atl31–1/atl6–1* was comparable to that of WT, suggested that the pale-green phenotype of *atl31–1/atl6–1* was not caused by high light-induced PSII photodamage. This finding, taken together with the normal Chl a/b ratio in *atl31–1/atl6–1*, suggests that the efficiency of photosynthesis in pale-green leaves of the double mutant was not damaged, although the photoreactive capacity could be reduced under ML conditions.

### Decreased *HEMA1* and *GLK1* expression in *atl31/atl6* pale-green leaves

**Fig 6:**
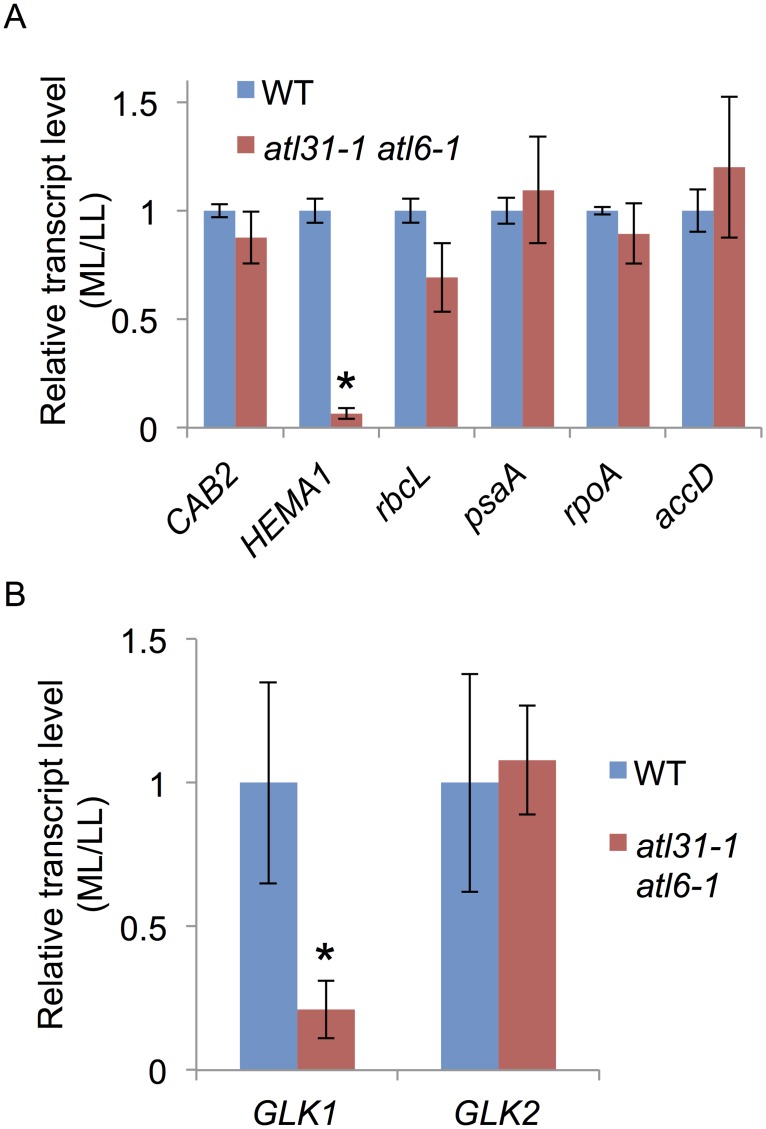
Expression of photosynthesis-related genes. Total RNA was extracted from 10-day-old WT and *atl31–1/atl6–1* plants grown under LL and ML conditions, and transcript levels of photosynthesis-related genes, *CAB2*, *HEMA1*, *rbcL*, *psaA*, *rpoA* and *accD* (A), *GLK1* and *GLK2* (B) were measured by quantitative real-time RT-PCR. *EF1α* was used as an internal control. The ratio of each transcript in *atl31–1/atl6–1* plants grown under ML vs. LL conditions were normalized relative to the ratio in WT plants. Error bars represent SD (n = 3). Asterisks indicate statistically significant differences between the mutants and WT plants by Student’s *t*-test (*, *P* < 0.001).

Using quantitative real-time RT-PCR, we assayed the level of transcripts of photosynthesis-related genes in WT and *atl31–1/atl6–1* (Fig. 6A). Genes assayed included the plastid-encoded RNA polymerase (PEP)-dependent *rbcL* and *psaA* genes; the nuclear-encoded-RNA polymerase (NEP)-dependent *rpoA* and *accD* genes; and the nuclear-encoded *CAB2* and *HEMA1* genes whose products are targeted to chloroplasts. Except for *HEMA1*, all of these genes in WT and *atl31–1/atl6–1* showed similar levels of expression in response to different light intensity conditions (Fig. 6A). In contrast, the expression of *HEMA1* in *atl31–1/atl6–1* was much lower under ML than under LL conditions compared with that in WT (Fig. 6A). Previously, *Golden 2-like* (*GLK*) transcription factors GLK1 and GLK2 were identified as positive regulator of nuclear photosynthetic genes including *HEMA1* through direct binding to their promoter sequences, resulting in pale-green phenotype of *glk* mutants [36]. Further analyses of *GLK1* and *GLK2* transcript levels revealed that *GLK1* was specifically down-regulated in *atl31–1/atl6–1* under ML condition as well as that of *HEMA1* (Fig. 6B). Since HEMA1 is the rate-limiting enzyme of tetrapyrrole biosynthesis [3], these results strongly suggest that the reduced *HEMA1* expression in *atl31–1/atl6–1* under ML conditions caused a down-regulation of Chl biosynthesis, manifesting as a pale-green phenotype.

### ALA application restored the normal phenotype of *atl31/atl6*

**Fig 7:**
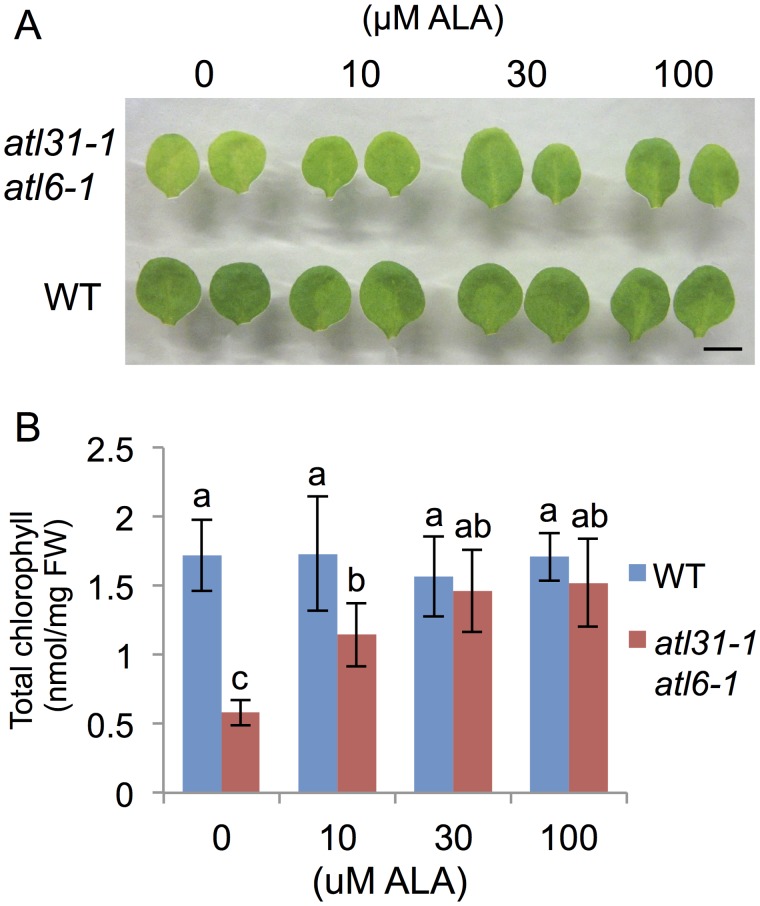
Restoration from pale-green phenotype of the *atl31–1 atl6–1* double mutant by exogenous ALA application. WT and *atl31–1/atl6–1* plants grown under ML conditions for 9 days were transplanted to medium with and without ALA and incubated for 3 days. (A) Photographs of representative first or second true leaves of each plant. Scale bar: 5 mm. (B) Amount of Chl in first or second true leaves of each plant. Error bars represent SD (n = 5). Statistical significance was determined by ANOVA, followed by post-hoc Tukey’s tests. Means that differed significantly (*P*<0.05) are indicated by different letters.

To confirm that Chl synthesis in the *atl31–1/atl6–1* double mutant was impaired at the rate-limiting step of ALA synthesis, and that limited ALA synthesis was responsible for the double mutant pale-green phenotype, we tested the effects of the addition of ALA. WT and *atl31–1/atl6–1* plants grown under ML conditions were transferred to medium containing 0, 10, 30 and 100 μM ALA nine days after germination and incubated for three days (Fig. 7A and 7B). The amount of Chl recovery in *atl31–1/atl6–1* was dependent on the concentration of ALA, with the highest doses showing leaves similar to those of WT plants (Fig. 7B). Enlarged photographs showed that newly emerging leaves near the petioles were the first to become fully green (S5 Fig.). Taken together of all results indicated that the pale-green phenotype of *atl31–1/atl6–1* was caused by inhibition of 5-aminolevulinic acid biosynthesis through the down-regulation of Chl biosynthesis-related genes expression.

## Discussion

The pale green phenotype of the *atl31/atl6* double mutant was apparently due to a reduction in *HEMA1* expression, followed by a reduction in Chl biosynthesis. Our expression analysis showed that *HEMA1* expression in *atl31/atl6* was markedly down-regulated under ML conditions, whereas the addition of exogenous ALA resulted in the dose-dependent recovery of WT phenotype.

The pale-green phenotype of *atl31/atl6* was similar to that of a *hema1* mutant recently characterized in Arabidopsis [12]. The plastid of this mutant, with 13% of the Chl present in WT, contains markedly lower numbers of thylakoids but a normal level of RbcL. Similarly, *atl31/atl6* had approximately 20% of the Chl in WT, with a notably reduced inner membrane structure under ML conditions and moderately reduced levels of RubisCO complex or RbcL. The similar results observed in the *hema1* mutant and *atl31/atl6* suggests that maturation of the inner membrane structure of both plastid is impaired by the reduction in Chl level.

ATL31 and ATL6 are involved in the regulation of *HEMA1* expression maybe through the regulation of *GLK1* expression. GLK genes encode a pair of partially redundant transcription factors that are required for the expression of nuclear photosynthesis related-genes including *HEMA1* [36,37]. Since GLK regulates expression of *Lhcb* gene family as well as *HEMA1*, decreased amounts of Lhcb1 protein in *atl31/atl6* double mutant was also consistent to this hypothesis. Present study revealed that the expression of *GLK1* was down-regulated in pale-green leaves whereas that of *GLK2* was not, ATL31 and ATL6 may regulate the expression of *GLK1* specifically by unknown mechanism.

ATL31 and ATL6 function in growth regulation in response to C/N-nutrient availability [15,16] and *atl31/atl6* double mutant showed pale-green phenotype due to decreased Chl biosynthesis (this study), implicating close relationship between C/N-nutrient status and Chl biosynthesis. Actually, the primary C and N metabolism interacted at Glu biosynthesis reaction through GS-GOGAT cycle [2,38], which provides Glu-tRNA, the substrate of ALA production by HEMA1. In addition, 14–3–3 proteins, the ubiquitination targets of ATL31 and ATL6, are involved in chloroplast biogenesis. The 14–3–3 protein family consists of 13 expressed isoforms, which bind to a wide variety of target proteins at phosphorylated motifs and function in multiple developmental processes by regulating their activity or sub-cellular localization [39,40]. Compared with WT, larger numbers of chloroplasts were found in the roots of 14–3–3μ and 14–3–3ν mutants, depending on the wavelength and intensity of light [41]. These findings, taken together with defects in light sensing and/or response displayed by mutants of 14–3–3μ and 14–3–3ν [42], suggest that these 14–3–3 proteins may be the key integrators between light signaling and chloroplast biogenesis. Since 14–3–3 proteins are also involved in light signaling that regulates *HEMA1* transcription [40], ATL31 and ATL6 may be involved in chloroplast biogenesis by regulating the stability of specific isoforms of 14–3–3 proteins in a light intensity-dependent manner. To date, however, the detailed mechanism of the relationship between 14–3–3 proteins and chloroplast biogenesis is still largely unknown.

Other ATL members may also participate in Chl biosynthesis. The pale-green phenotype of *atl31–1/atl6–1* was specific to true leaves and transient in younger developmental stages. In addition, the down-regulation of *HEMA1* expression only occurred early during true leaf development, followed by a deficiency in plastid biogenesis. These results suggest that ATL31 and ATL6 regulate *HEMA1* expression in true leaves of young plants early during development. Since the ATL family in Arabidopsis consists of 91 members [14], other members of this family could be participating in Chl biosynthesis at different developmental stages or in different tissues.

Although, determination of the detailed molecular mechanism by which ATL31 and ATL6 regulate *HEMA1* expression is still unknown, further investigation of ATL31 and ATL6 provide us a new aspect of Chl biosynthesis regulation.

## Supporting Information

S1 Fig *atl31–1/atl6–1* double mutants show no obvious difference at mature stage. Photographs of representative *atl31–1/atl6–1* (A) and WT (B) plants at 40 days after germination (DAG). Scale bar: 5 cm.
(TIFF)

S2 Fig Single mutants of *ATL31* or *ATL6* and overexpressors of these proteins did not show a pale-green phenotype. (A) Photographs of representative plants of 2-week mutant plants grown under ML or LL conditions. Scale bar: 5 mm. (B) and (C) Chl amounts in the first or second leaves of each mutant plants grown under ML (B) and LL (C). Error bars represent SD (n = 5). Statistical significance was determined by ANOVA, followed by post-hoc Tukey’s tests. Means that differed significantly (*P*<0.05) are indicated by different letters.
(TIFF)

S3 Fig Manifestation of the pale-green phenotype in the double mutant *atl31–1/atl6–2* and after transfection of *ATL31/6* RNAi. (A) Schematic diagram of the *ATL6* gene structure and the location of T-DNA insertions in the *atl6–2* (SALK_134489) mutant. *ATL6* consists of a single exon (black box) with 5’- and 3’-UTRs (white boxes). (B) RT-PCR analysis of *ATL6* mRNA transcript levels in the *atl6–2* mutant Total RNA from 4-week-old vegetative shoot tissues were analyzed. *EF1α*, control expressed gene. (C) Photographs of representative *atl31–1/atl6–2* and *ATL31/6* RNAi plants grown under ML conditions for 8 days. Scale bar: 1 mm. (D) RT-PCR analysis of *ATL31* and *ATL6* mRNA transcript levels in the *ATL31/6* RNAi#4. Total RNA from 4-week-old vegetative shoot tissues were analyzed. *EF1α*, control expressed gene.
(TIFF)

S4 Fig Plastid ultrastructure. Transmission electron microscopic images of first or second true leaves of 2-week old plants *atl31–1/atl6–1* (A-D) and WT (E-H) plants grown under ML (A-C and E-G) and LL (D and H) conditions. The section of each sample was indicated by a red line on the left below representative leaves. Scale bar: 1 μm.
(TIFF)

S5 Fig Restoration from pale-green phenotype of the *atl31–1 atl6–1* double mutant by exogenous ALA application. Plants with the *atl31–1/atl6–1* double mutation grown under ML conditions for 9 days were transplanted to ALA containing medium and incubated for 3 days. Photographs of representative plants are shown in upper panels. Middle panels show enlarged first or second leaves and bottom panels show newly emerged leaves Scale bar: 5 mm.
(TIFF)

S1 Table Primer sequences for vector constructs and gene expression assays. (TIFF)
