# Supplementary figures and images for: Pale-Green Phenotype of atl31 atl6 Double Mutant Leaves Is Caused by Disruption of 5-Aminolevulinic Acid Biosynthesis in Arabidopsis thaliana
Source: PLoS One. 2015 Feb 23;10(2):e0117662. doi: 10.1371/journal.pone.0117662 (PMC4338271; doi:10.1371/journal.pone.0117662)

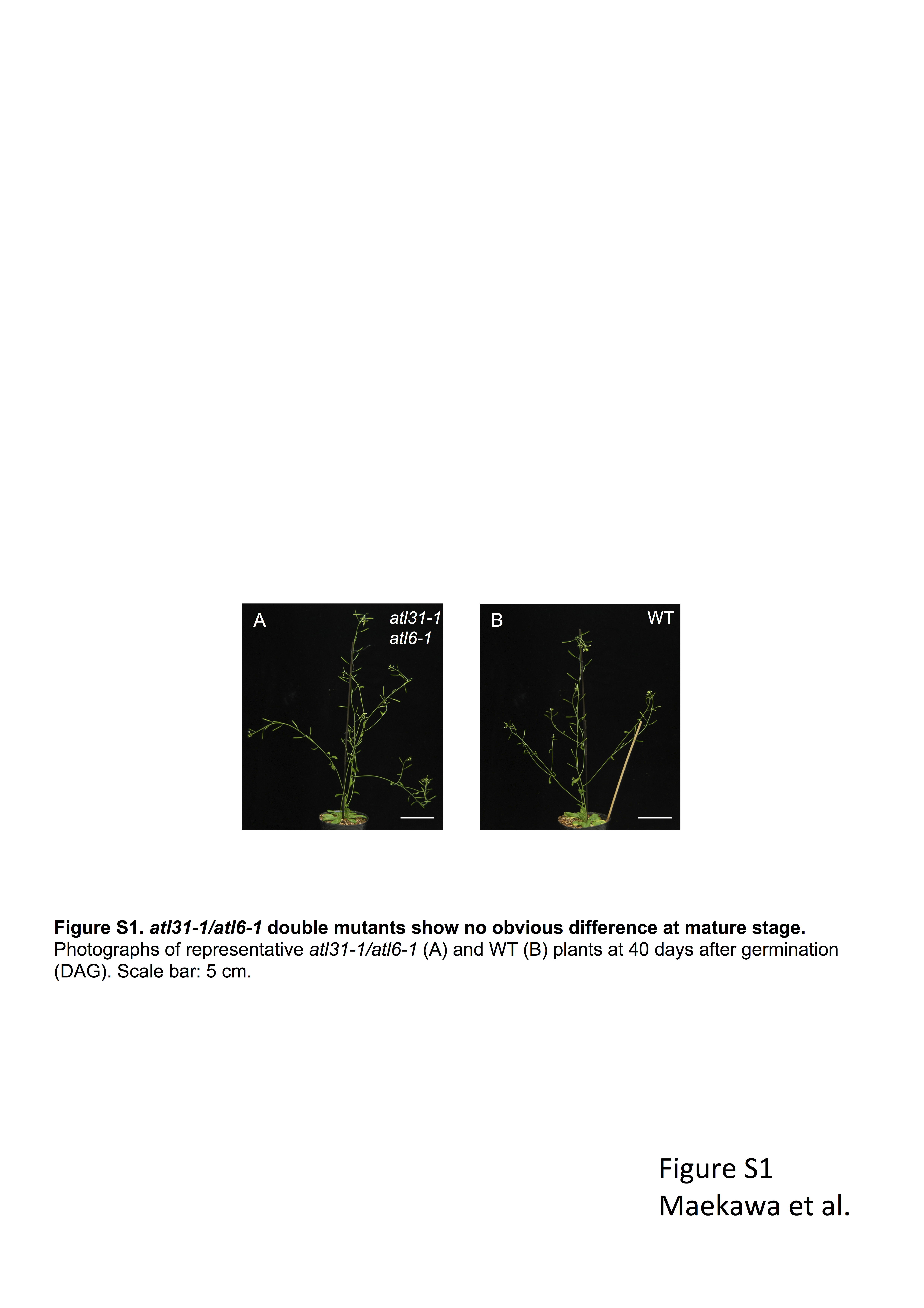

Supplement: S1 Fig — Photographs of representative atl31–1/atl6–1 (A) and WT (B) plants at 40 days after germination (DAG). Scale bar: 5 cm. (TIFF) [file pone.0117662.s001.tiff]

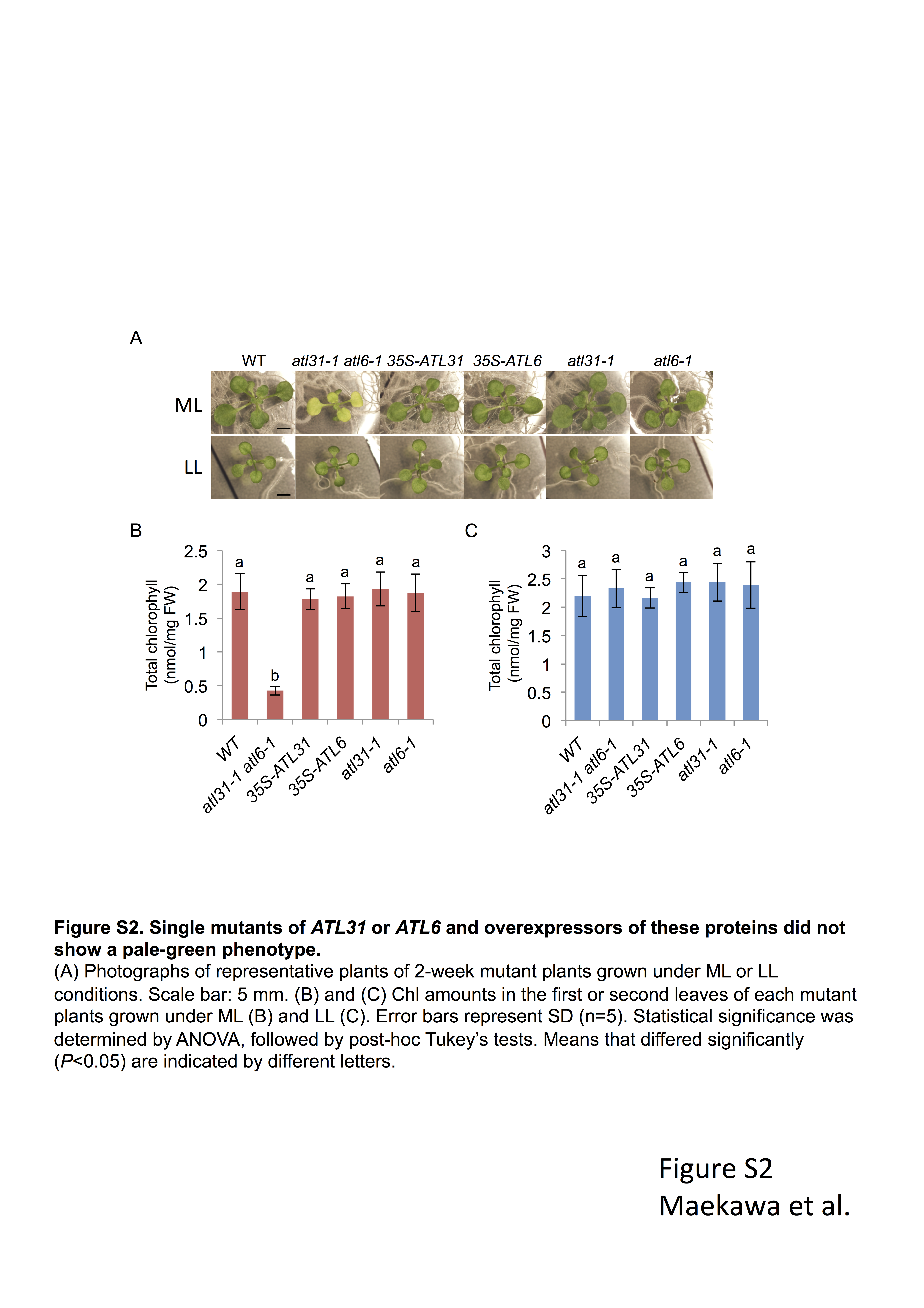

Supplement: S2 Fig — (A) Photographs of representative plants of 2-week mutant plants grown under ML or LL conditions. Scale bar: 5 mm. (B) and (C) Chl amounts in the first or second leaves of each mutant plants grown under ML (B) and LL (C). Error bars represent SD (n = 5). Statistical significance was determined by ANOVA, followed by post-hoc Tukey’s tests. Means that differed significantly (P<0.05) are indicated by different letters. (TIFF) [file pone.0117662.s002.tiff]

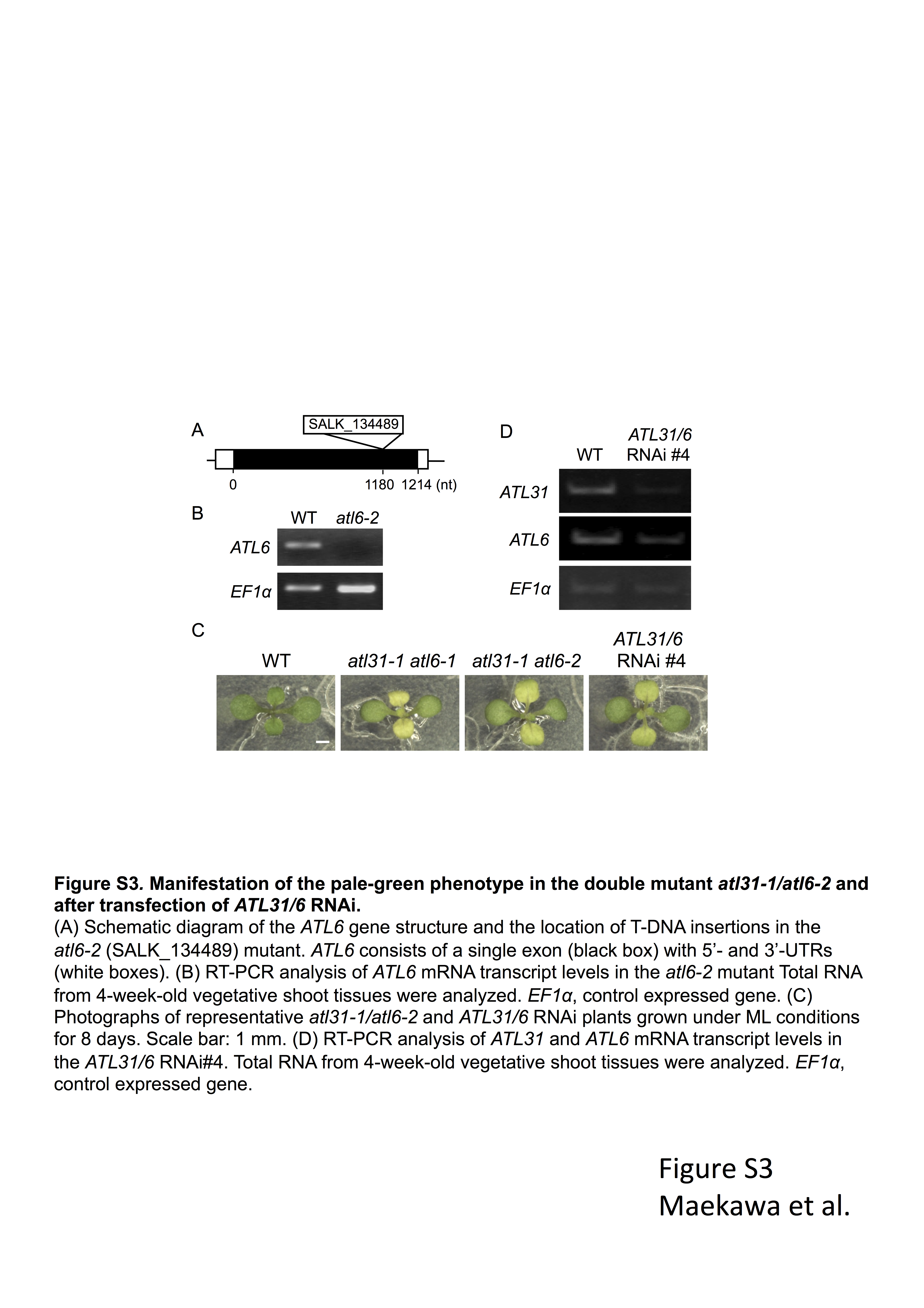

Supplement: S3 Fig — (A) Schematic diagram of the ATL6 gene structure and the location of T-DNA insertions in the atl6–2 (SALK_134489) mutant. ATL6 consists of a single exon (black box) with 5’- and 3’-UTRs (white boxes). (B) RT-PCR analysis of ATL6 mRNA transcript levels in the atl6–2 mutant Total RNA from 4-week-old vegetative shoot tissues were analyzed. EF1α, control expressed gene. (C) Photographs of representative atl31–1/atl6–2 and ATL31/6 RNAi plants grown under ML conditions for 8 days. Scale bar: 1 mm. (D) RT-PCR analysis of ATL31 and ATL6 mRNA transcript levels in the ATL31/6 RNAi#4. Total RNA from 4-week-old vegetative shoot tissues were analyzed. EF1α, control expressed gene. (TIFF) [file pone.0117662.s003.tiff]

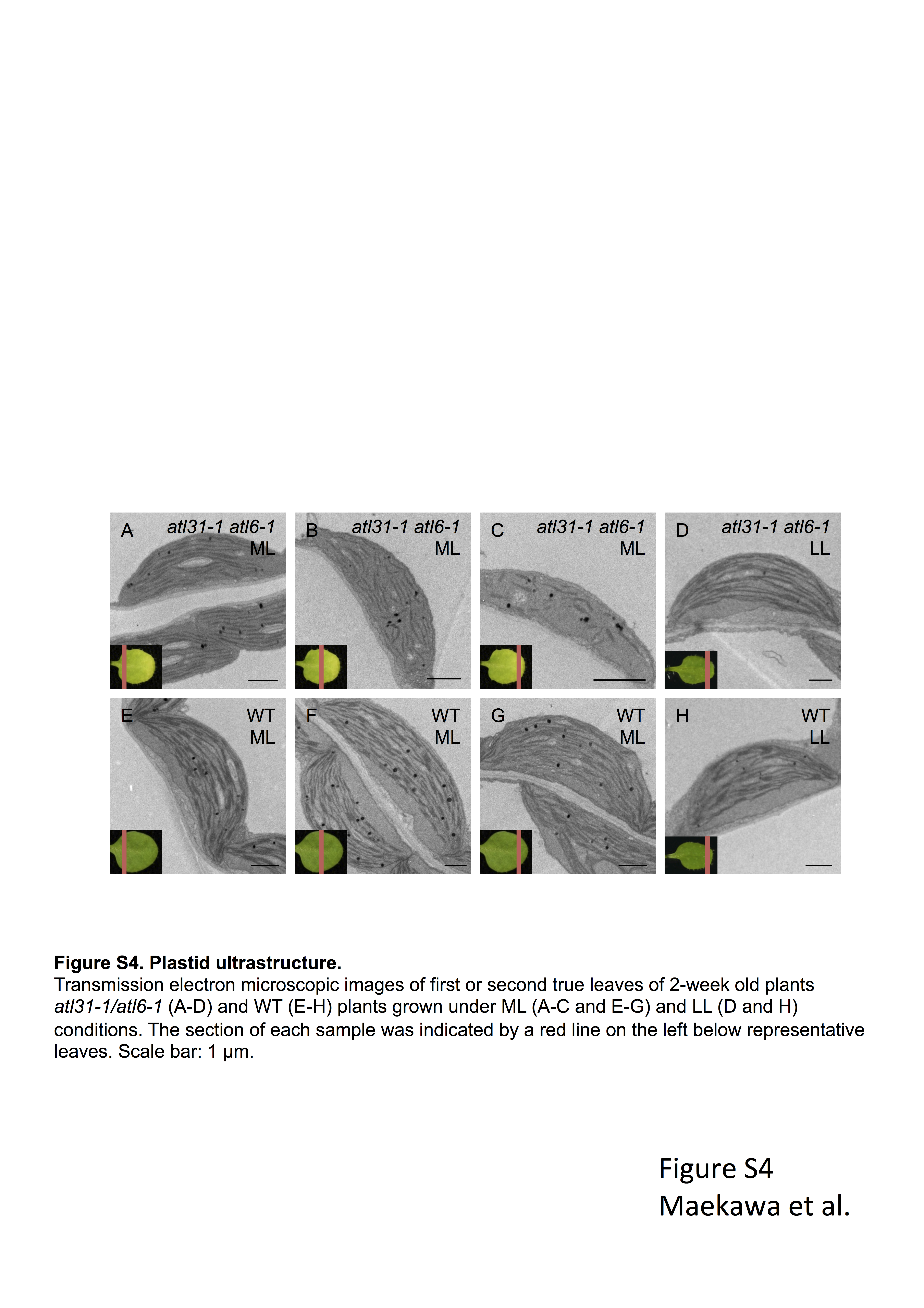

Supplement: S4 Fig — Transmission electron microscopic images of first or second true leaves of 2-week old plants atl31–1/atl6–1 (A-D) and WT (E-H) plants grown under ML (A-C and E-G) and LL (D and H) conditions. The section of each sample was indicated by a red line on the left below representative leaves. Scale bar: 1 μm. (TIFF) [file pone.0117662.s004.tiff]

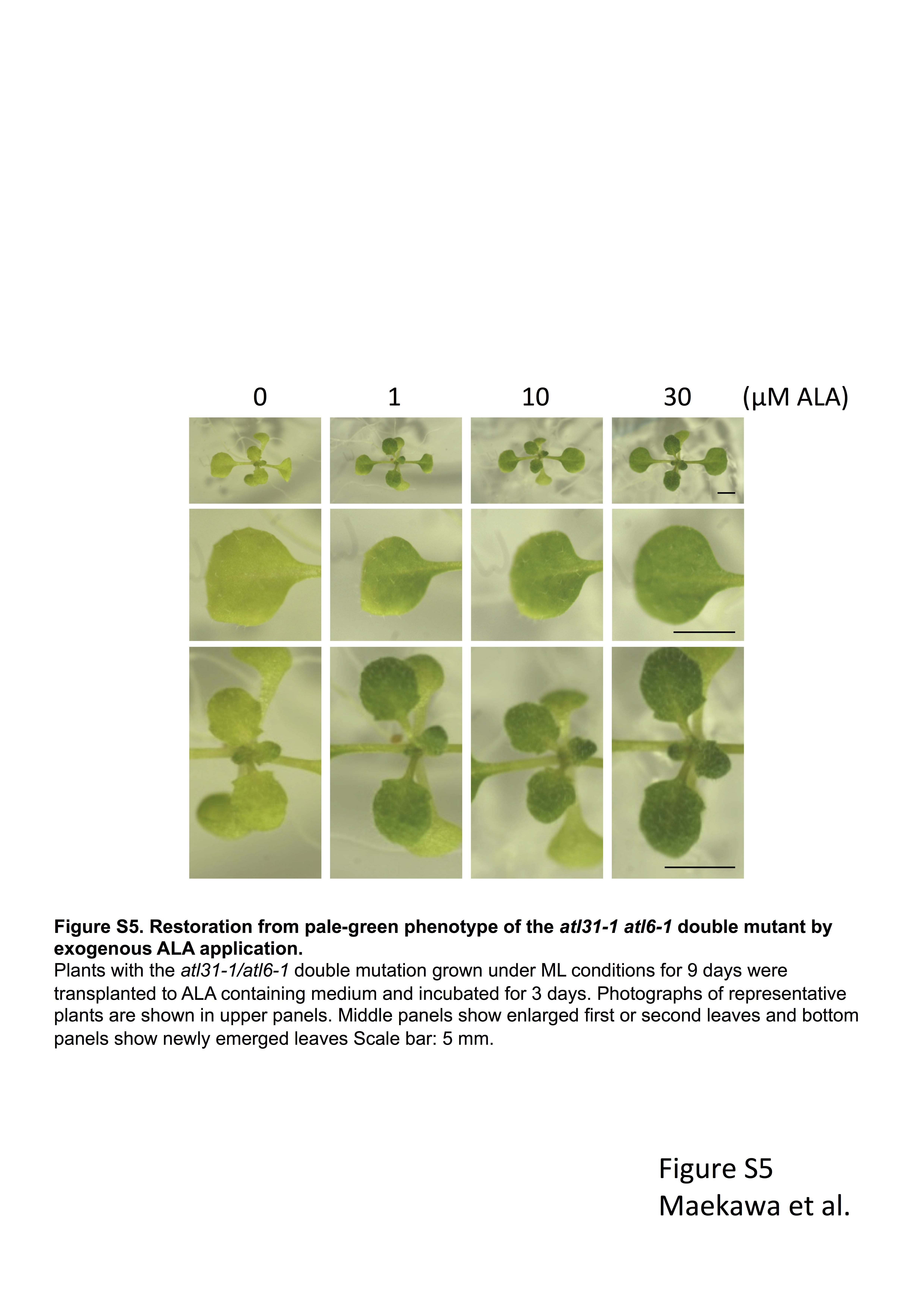

Supplement: S5 Fig — Plants with the atl31–1/atl6–1 double mutation grown under ML conditions for 9 days were transplanted to ALA containing medium and incubated for 3 days. Photographs of representative plants are shown in upper panels. Middle panels show enlarged first or second leaves and bottom panels show newly emerged leaves Scale bar: 5 mm. (TIFF) [file pone.0117662.s005.tiff]

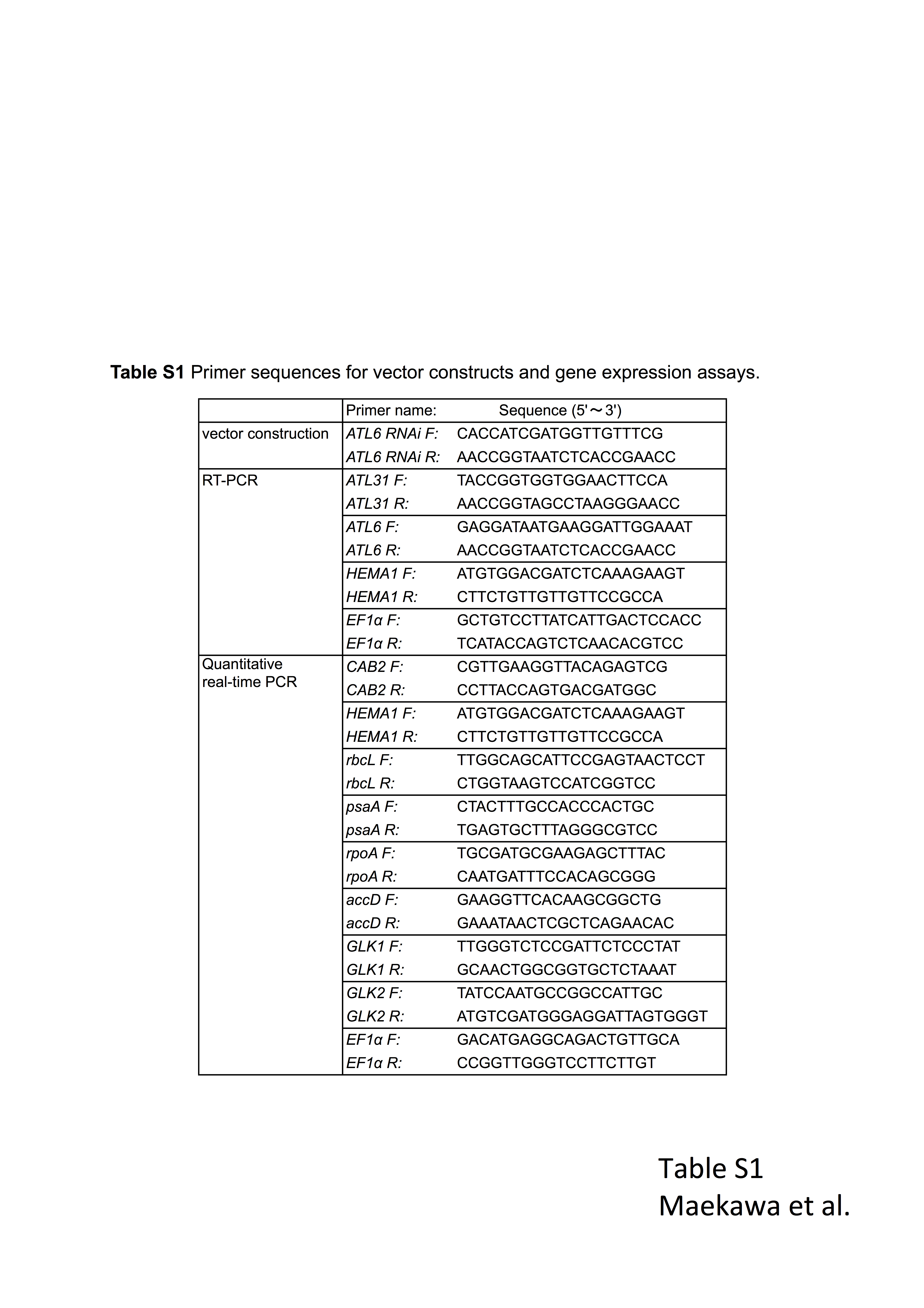

Supplement: S1 Table — (TIFF) [file pone.0117662.s006.tiff]
